# The Prevalence of Migraine With Anxiety Among Genders

**DOI:** 10.3389/fneur.2020.569405

**Published:** 2020-10-26

**Authors:** Leila Karimi, Sheila Gillard Crewther, Tissa Wijeratne, Andrew E. Evans, Leila Afshari, Hanan Khalil

**Affiliations:** ^1^School of Psychology and Public Health, La Trobe University, Melbourne, VIC, Australia; ^2^Faculty of Social and Political Sciences, Ivane Javakhishvili Tbilisi State University, Tbilisi, Georgia; ^3^Department of Neurology, AIMSS, Level Three, WHCRE, Sunshine Hospital, University of Melbourne, St Albans, VIC, Australia; ^4^Department of Medicine, Faculty of Medicine, University of Rajarata, Anuradhapura, Sri Lanka; ^5^Royal Melbourne Hospital, Melbourne, VIC, Australia; ^6^School of Business, La Trobe University, Melbourne, VIC, Australia

**Keywords:** anxiety, migraine, gender difference, prevalence, systematic review

## Abstract

**Objective:** The aims of the present systematic review were to explore the prevalence of migraine with anxiety exclusively and determine if and why there are likely to be differences across genders.

**Introduction:** Migraine is a very common neurological disorder and cause of productive disability worldwide that is more frequent in women of childbearing age than males. Previous studies have frequently demonstrated comorbidity of migraine and other psychiatric disorders. Although the prevalence of migraine across gender is well-established there are few if any systematic reviews on the prevalence of migraine comorbidity with anxiety cross-genders.

**Methods:** The present systematic review included prevalence studies, clinic-based and cohort studies that reported the frequency of migraine with anxiety within the study sample. Eleven studies were included in the review after screening by two independent reviewers. Studies included participants who were 16 years and older diagnosed with migraine.

**Results:** The main findings of this review indicated that anxiety is a major comorbidity of migraine worldwide, with a wide range (16–83%) of prevalence and a mean of ~43% of patients experiencing comorbid symptoms. Subjective anxiety symptoms appear to be greater among males with migraine than females which could be attributable to both environmental and/or hormonal and genetic predispositions.

**Conclusions:** The results reemphasize the high prevalence of migraine and comorbid anxiety symptoms worldwide while showing that although migraine is far more prevalent among women in general co-morbidity of migraine with anxiety unfolds a different gender difference. The results highlight the significance of exploring the impact of existing and pre-existing comorbid conditions of patients with migraines and further consideration into their diagnostic and treatment strategies.

## Introduction

Migraine is often cited as one of the most prevalent disorders globally (1) though there is a large regional variation. The lowest prevalence is reported in Africa, followed by Asian countries, higher in European countries, North America and highest reported in Australia (World Health Organization (WHO), 2020). About 30% of people with headache are diagnosed with migraine which is typically conceptualized as a chronic disorder with episodic attacks (2) and where subtypes are defined either as episodic migraine (EM) or chronic migraine (CM) depending on the frequency of cooccurrence with EM occurring on <15 days a month (EM) and CM occurring with greater frequency per days of the month. EM often develops into CM, and this transition is termed transformation, chronification, or progression (3).

In Australia alone, migraine is one the main causes of disability, with ~5 million individuals suffering from migraine, representing ~20% of the nation's population (4). The prevalence of migraine increases for individuals aged between 12 and 40 of both sexes, and results in a substantial loss of productivity due to missing days of school or work, and mostly requiring bed rest (4, 5). Indeed, majority of migraine sufferers are of working age, and 71% are women between the ages of puberty and menopause (6).

The cost of migraine in Australia in 2018 has been assessed recently at around $35.7 billion (7). The Global Burden of Diseases (GBD), disease burden is estimated in disability-adjusted life-years (DALYs), which are “the total years of life lost to premature mortality and years of life lived with disability.” Years lived with disability for headache disorders “are calculated from its prevalence and the mean time patients spend with the headache, multiplied by the associated disability weight.” Indeed, the migraine disability weight compared to a healthy person based on this calculation was estimated as 43.4% (8). Despite the substantial health burden of migraine, it is neither diagnosed adequately worldwide (3, 4, 7) nor is its etiology well-understood nor is research well-funded anywhere. Indeed, the topic has largely been ignored with only a small number of publications in the neurological literature dealing with the association of migraine and the commonest psychiatric disorder Generalized Anxiety Disorder (GAD) though more are available studies relating to comorbidity of migraine and depression (9, 10).

For example, a 2007 Canadian study has found that panic disorder was almost three times more common among migraineurs than others (10). Other studies demonstrated that anxiety disorders are more prevalent among people with migraine compared to non-migraineurs (11–14) and not surprisingly other studies have reported that the prevalence of anxiety increases significantly with the frequency of migraine attack episodes (15). Conversely, stress has been identified as a trigger for nearly 75% of migraine attacks (16) indicating that the relationship between anxiety and migraine is a complex one.

Furthermore, migraine seems to be more prevalent among female than male population (5, 16). The prevalence rate in some studies reported among males compared to females ranging from 5 to 8% and 11 to 16% in order (17) presumably due to estrogen hormone fluctuations during the periodic cycle. As suggested in “Estrogen withdrawal hypothesis” of Somerville (17), decreasing estrogen levels, for example before menstruation, may trigger migraine attacks (17). Similar gender based observations were made by Victor et al. (18) in their national epidemiological health survey in US. Based on a more recent systematic analysis for the Global Burden of Disease Study 2016 (8) age-standardized prevalence rate of migraine estimated around 14% worldwide with the prevalence rate much higher for females (19%) compared to males (10%).

Although the prevalence of migraine across gender is well-established in the previous studies, in the authors knowledge there is no systematic review on the prevalence of migraine comorbidity with anxiety cross-genders. The aims of the present systematic review were to explore the prevalence of migraine with anxiety and determine if there are differences across genders.

## Review

### Methods

#### Eligibility Criteria

The review considered prevalence studies both clinic-based and cohort studies that reported the frequency of comorbid migraine with anxiety within the study sample. Studies with participants aged 16 years and older diagnosed with migraine clinically by a medical practitioner or according with The International Classification of Headache Disorders, 2nd/3rd edition (ICHD-2/3) were included. Studies were excluded from the review if (a) no data were reported on the prevalence or frequency of migraine with anxiety; (b) if the sample investigated included children or adolescents aged <16 years; or (c) if an abstract was not be available in English. The main outcomes of interest considered for this review were any report of anxiety and prevalence of migraine with anxiety exclusively.

#### Data Sources

A systematic search was conducted on 30/12/2019 using the following electronic databases, Medline, EMBASE (Ovid), Cochrane, and PubMed. Studies published as far back as possible were considered for inclusion in the review. Searches was done by migraine, anxiety and epidemiology keywords as outlined in Table 1.

**Table 1:** Key terms used in the search.

**Migraine**	**Anxiety**
Migraine disorders (Mesh) Tension headache (Mesh) Cluster headache (Mesh) Tension adj2 headache Chronic adj4 headache Chronic adj migraine^*^ Migraine^*^	Anxiety Anxieties GAD Panic^*^ Neurotic Neuros# Anxiety panic anxiety Disorders Panic disorders Neurotic Disorders

#### Study Selection

After completing the search stage, all the identified citations were imported into EndNote (version X9.2). All the duplicate citations were removed. The abstracts were assessed by two independent reviewers (LK and HK). Full text citations were imported into the JBI Sumari. The full text in the last step were screened by two independent reviewers (LK and HK).

#### Quality Assessment

The risk of bias was evaluated with the appraisal tools from Joanna Briggs Institute critical for each study types (prevalence, cohort, and cross-sectional studies) (19, 20). The results are presented separately at Tables 2–4.

**Table 2:** Risk of bias in prevalence studies.

**Studies**	**Was the sample frame appropriate to address the target population?**	**Were study participants sampled in an appropriate way?**	**Was the sample size adequate?**	**Were the study subjects and the setting described in detail?**	**Was the data analysis conducted with sufficient coverage of the identified sample?**	**Were valid methods used for the identification of the condition?**	**Was the condition measured in a standard, reliable way for all participants**	**Was there appropriate statistical analysis?**	**Was the responses rate adequate, and if not, was the low response rate managed appropriately?**
Oh et al. (21)	Yes	Yes	No	Yes	Yes	Yes	Yes	Yes	No
Rammohan et al. (14)	Yes	Yes	No	Unclear	Yes	Yes	Yes	Yes	Unclear
Senaratne et al. (22)	Yes	Yes	Unclear	Yes	Yes	Yes	Yes	Yes	Yes
Victor et al. (18)	Yes	Yes	Yes	Yes	Yes	Yes	Yes	Yes	Yes
Yong et al. (23)	Yes	Yes	No	Yes	Yes	Yes	Yes	Yes	Yes

**Table 3:** Risk of bias in Cross sectional studies.

**Studies**	**Were the criteria for inclusion in the sample clearly defined?**	**Were the study subjects and the setting described in detail?**	**Was the exposure measured in a valid and reliable way?**	**Were objective, standard criteria used for measurement of the condition?**	**Were confounding factors identified?**	**Were strategies to deal with confounding factors stated?**	**Were the outcomes measured in a valid and reliable way?**	**Was appropriate statistical analysis used?**
Mercante et al. (24)	Yes	Yes	Yes	Yes	Unclear	Unclear	Yes	Yes
Lampl et al. (25)	Yes	Yes	Yes	Yes	Yes	Unclear	Yes	Yes
Peres et al. (26)	Yes	Yes	Yes	Yes	Unclear	Unclear	Yes	Yes
Song et al. (27)	Yes	Yes	Yes	Yes	Unclear	Unclear	Yes	Yes
Wachholtz et al. (28)	Yes	Yes	Yes	Yes	Unclear	Unclear	Yes	Yes

**Table 4:** Risk of bias in cohort studies.

**Studies**	**Were the two groups similar and recruited from the same population?**	**Were the exposures measured similarly to assign people to both exposed and unexposed groups?**	**Was the exposure measured in a valid and reliable way?**	**Were confounding factors identified?**	**Were strategies to deal with confounding factors stated?**	**Were the groups/participants free of the outcome at the start of the study (or at the moment of exposure)?**	**Were the outcomes measured in a valid and reliable way?**	**Was the follow up time reported and sufficient to be long enough for outcomes to occur?**	**Were strategies to address incomplete follow up utilized?**	**Was appropriate statistical analysis used?**
Karakurum et al. (29)	Yes	Yes	Yes	Unclear	Unclear	Unclear	Yes	Unclear	Unclear	Yes

#### Data Extraction

Data extraction included publication information, methods, objectives, migraine definition, study population, and other main demographic data. Prevalence, percentage and/or incidence estimates were pulled out for population studies. Confidence interval of 95% were reported/calculated for the prevalence rate. For clinical based/cohort studies, the migraine frequency, characteristics, and disease burden were extracted.

## Results

### Study Inclusion

**Figure 1:**
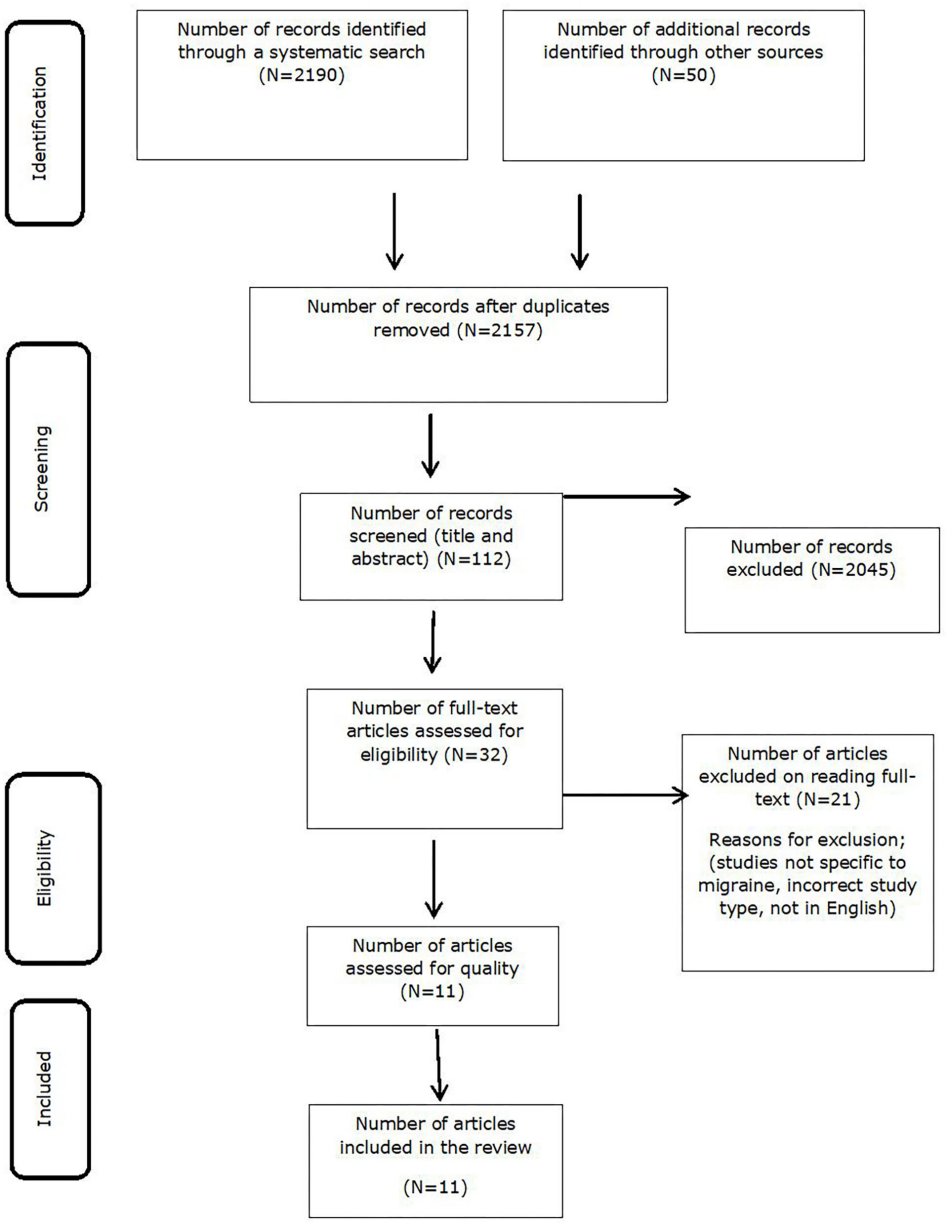
PRISMA.

The search identified 2,190 studies adding to 50 studies that were identified by hand searching (through Google scholar, ResearchGate, and other internet sources). In total 2,240 citations were identified in which 83 were duplicates and were removed at the next step ended up with 2,157 unique citations. During the screening for abstract and title, 112 studies were selected; further full text review identified 32 studies eligible to be included. In the final full text review 21 studies were removed at the last assessment step as they did not meet inclusion criteria as set above. The 11 studies included in the final systematic review as shown in Figure 1.

### Quality Assessments

The risk of bias in the studies were evaluated with the Joanna Briggs Institute critical appraisal tools for prevalence, cohort, and cross-sectional studies as shown in Tables 2–4 (19, 20) and are detailed below according to study type.

#### Prevalence Studies

There was a total of five prevalence studies (14, 18, 21, 22). Only one study (23) fulfilled all the requirements for a high-quality study. All included studies had adequate sampling, valid methods for identifying the condition and data analysis. Small sample size was the major limitation in three studies (Table 5).

**Table 5:** Characteristics of Included Studies—Prevalence, cohort, and cross-sectional studies.

**Prevalence**							
**Country/author**	**Methods (data collection procedure)**	**Sample size**	**Age (years) Range/mean (SD)**	**Migraine diagnosis**	**Anxiety criteria**	**Overall migraine Prevalence 95% N CI**	**Prevalence of migraine with anxiety %- N/Odds ratio (CI) vs. no headache or healthy participants**
South America							75%−192
Brazil/Mercante et al. (24). South America	The Anxiety Disorders Program of the Institute of Psychiatry	60 F: 39 M: 21	19–70	ICHD-II	GAD	66.6%−20 F: 69.5%−16 M: 57.1%−4	66.7%−20 F: 69.5%−16 M: 57.1%−4
Brazil/Peres et al. (26). South America	Primary care self-administered questionnaire	782	34.2 (6.3)	Self-reported ICHD-II	GAD-7 (anxiety)	213 migraine	83%−177
North America							52%−2,699
Canada/Senaratne et al. (22). North America	Outpatient anxiety clinic- computer-assisted telephone interview (CATI)	206 F: 144 M: 62	37.8 (12.9)	IHS	GAD	67%−138 F: 70.8%−102 M: 58.1%−36	58.5%−62 No significant effects were seen for gender
US/Victor et al. (18). North America	Epidemiological national survey	30,790 F: 17,394 M: 13,396	43.6	Self-reported medical diagnosis of migraine	Self-reported anxious symptomology [25]	15.2%−4,680 (14.7, 15.7) F: 20.5%−3,565(19.7, 21.3) M: 9.4%−1,259 (8.8, 10.0)	2.30 (2.09, 2.52) 43.5%−2,035 F: −93% M: 142%
US/Wachholtz et al. (28). North America	The online chronic migraine population	4,787 F: 4,554 (95.1%) M: 233 (4.9%)	18–65	Self-reported medical diagnosis of migraine	Not mentioned	100%	56.4%−2,699
Asia							38%−205
Turkey/Karakurum et al. (29). Asia	n/c	87	CM: 32.1 (10.4) EM: 32.6 (9.7)	IHS	Hamilton Anxiety Scale (HAS)	100%	75.6%−28
Korea/Oh et al. (21). Asia	Primary care-population based surveys	2,762 F: 1,385 M: 1,377	19–69	ICHD-2	Goldberg Anxiety Scale [13]	5.4%−147 F: 8.0%−111 (6.6–9.4) M: 2.6%−36 (1.8–3.5)	30.1%−45 F: 23.1%−34 M: 30.5%−11
India/Rammohan et al. (14). Asia	Migraine patients of the Neurology Outpatient Department	133 F: 103(78%)	34.13 (8.49)	ICHD-3	The Hospital Anxiety Scale (HADS-A)	100%−133	16.54%−22 No association was found between gender.
China/Yong et al. (23) Asia	Headache outpatient clinic	185 F: 144 (81.8%) M: 32 (18.2%)	14–63 39.1 (11.6)	ICHD-2	HADS	95%−176	38.1%−67 F: 88.0%−59 M: 11.9%−8
Korea/Song et al. (27). Asia	Primary care-population based structured interviews	2,695 F: 1,350 (50.7) M: 345 (49.3)	19–69	ICHD-3	State-Trait Anxiety Inventory (STAI)	5.3%−143 (4.5–6.2) F: 7.9%−107 (6.5–9.4) M: 2.7%−36 (1.8–3.5)	30.1%−43
Europe							19%−454
European union countries/Lampl et al. (25). European	Primary care-population based surveys	6,624 F: 3,655 M: 2,969	42.1 (12.9)	ICHD-2	HADS	35.9%−2,375 (34.7–37.1) F: 43.1%−1,575 (41.5–44.7) M: 26.9%−799 (25.3–28.5)	19.1%−454 (17.5–20.7) F: 140% M: 320%

#### Cross Sectional Studies

There was a total of five cross sectional studies (24–27). All studies had appropriate sampling, adequate description of study subjects and statistical analysis. Only one study (25) identified confounding factors but did not adjust for them. Confounding factors were not identified in any of the other four studies considered as limitations in interpretation of these studies.

#### Cohort Studies

There was only one cohort study (29) found that met criteria for inclusion in the review. The authors described both the exposure and the outcome assessed in a valid and reliable method, follow up time, covariates and methods to control them were not listed. Appropriate statistical analysis was reported.

### Study Characteristics

The characteristics of systematic review on the prevalence of migraine with anxiety are presented in Table 5. The age of the participants reported in the studies were on average between 34 and 44 years old of both genders. There was a consistency in diagnosis of migraine and almost in all the studies, except for one (21), migraine was diagnosed using ICHD-II/III. Contrary to migraine diagnosis, different scales were used for assessing or screening anxiety. Three studies used Hospital Anxiety Scale (HADS) (22) one study used Goldberg Anxiety Scale (13, 18), One study used State-Trait Anxiety Inventory (STAI) (23), one used self-reported anxious symptomology (21, 25), three studies used Goldberg Anxiety Scale (GAD) (24–26). One study did not mention their anxiety assessment tool (16). Studies were conducted in ten European union countries, India, China, USA, Korea, Brazil, Turkey, and Canada.

### Migraine and Anxiety Comorbidity

In a small study of migraine outpatient clinic in India (13), the prevalence of migraine with anxiety was reported to be as low as 16%, which is much lower than other studies reported in this systematic review. On the other hand, the Eurolight project that was a large population study across ten European Union countries, migraine prevalence with anxiety was observed at 19%. In this large study a range of questionnaires including HADS were used to assess anxiety among adult migraineurs.

Conversely, several studies internationally have described a much higher comorbid prevalence of migraine and anxiety. Two Brazilian studies (24) reported comorbid incidences of 83 and 67%, while a Turkish study by Karakurum et al. (29) reported 76% and the Canadian study of Senaratne et al. (22) found 58% incidences.

Moderate prevalence (43–56%) was reported in the US population studies of Victor et al. (18) and Wachholtz et al. (28). Similarly, among the Chinese migraineurs, 38% of anxiety was reported (27). In both large population-based studies in Korea (18), almost one-third of migraineurs were suffering from anxiety.

In summary, the prevalence of migraine with anxiety among migraine sufferers of all eleven studies ranging from 16 to 83% with a median of 43%. Lowest prevalence reported in India (16%), ten European Union countries (19%). When comparing the prevalence of migraine with anxiety based on regions, the highest prevalence reported in studies of South America (75%) followed by North America (52%), Asia (38%), and lowest in Europe (19%).

### Gender Differences in the Prevalence of Migraine With Anxiety

In almost all the studies, the prevalence of female with migraine is higher than males. However, when the comorbidity of migraine with anxiety is taken into account, the prevalence is almost the opposite. In the seven studies that reported enough information for drawing a conclusion on the prevalence of migraine with anxiety among genders, more than half of the studies (57%) reported a higher likelihood of males with anxiety among migraineurs compared to females. The Eurolight project involved patients recruited from primary care in ten European countries and found that the prevalence of migraine with anxiety among men (320%) was more than double that of female migraineurs (140%) when compared against no headache group. Similar results were reported in the Korean population study (21) where males prevalence of migraine with anxiety (30%) was slightly higher than females (23%).

In the US population studies (18) similar trends were reported where the prevalence of migraine with anxiety among men (142%) was higher than women (93%).

Only two studies in China (23) and Brazil (24) found a higher migraine prevalence with anxiety in females (88 and 69% in order) compared to the males (12 and 57% in order). However, it should be noted that both of these recruited participants from the headache clinic. Two studies did not find any significant differences between gender (14) 2010, and four did not report data on gender differences (26–29).

## Discussion

The main aims of the systematic review were to explore the prevalence of migraine comorbidity with anxiety exclusively as one of the most common disorders among migraineurs and to further explore the gender differences in such comorbidity.

Eleven studies were included in the systematic review on the prevalence of migraine with anxiety among migraineurs. Studies were population-based, clinical-based or cohort studies of different regions (Canada, US, Turkey, China, India, Korea, Europe, and Brazil).

The current review found that the prevalence of migraine with anxiety ranged between 16 and 83% with a median of 43%. As per population-based studies in this systematic review, the incidence of migraine with anxiety is prevalent among one-third of migraineurs which is consistent with many of the previous studies (10, 30, 31).

In the present review, findings showed that the prevalence of females with migraine was significantly higher than males which is well-established in the previous studies (22, 24, 26). However, the prevalence of migraine with anxiety was much higher among men compared to women. Despite different study settings, regions, age and other individual characteristics, evidence of greater prevalence in males was consistent in 9/11 (i.e., the majority of the studies included in the review). This is the first study to report on this gender difference. In the presence of such gender differences, and the fact that so many women experience migraine at the same time in their menstrual cycle and come to expect it as a monthly occurrence it is possible that the onset of migraine is half expected very month and so less anxiety inducing. By comparison males may find migraine socially unusual and hence more ‘worrying'. Interestingly migraine and anxiety comorbidity in males is reported to be accompanied by low testosterone levels (32). Gender differences have been documented in chronic conditions such as chronic kidney diseases, obstructive pulmonary diseases and others. This is also consistent with the Health lifestyle theory which suggests that variables such as gender, age and social networks dispose the individuals to certain practices that make them more likely to develop a condition than others (33, 34).

While exploring the underlying causes or direction of such complex relationship is difficult to explain in the existing studies, the results of the review suggest that clinicians need to be aware of the high prevalence of anxiety and migraine in their clinical settings. Early identification of comorbid conditions has the potential for better prognosis and improvement of patients outcomes (35). The routine screening of patients with migraine for anxiety or any other psychiatric/mood disorder is highly recommended in clinical settings.

The HADS was the most commonly used scale in the migraine studies. HADS is a validated, convenient scale for screening anxiety since it emphasizes the subjective indicators of anxiety as well as somatic symptoms and depression. However, HADS is more of a screening tool for physiological measures of anxiety rather than one for clinical diagnosis of anxiety and hence might lead to the underestimation of the prevalence of anxiety (36).

Unfortunately, the majority of anxiety scales are subjective and there is no objective unified scale currently available for screening or diagnosis of anxiety highlighting the need for a unified, objective, biological assessment of anxiety to ensure transparency of reporting and conclusive diagnosis of migraine.

### Limitations

The current systematic review is limited in generalizability by the relative scarcity of studies and rigor of the analyses currently available. Half of the studies we have included are cross-sectional. Four studies do not include a reference to potential confounding factors. Secondly, around half of the studies in this review were primarily prevalence studies and among these, sample size was a further limitation. Lastly, the diversity of screening tools used to diagnose anxiety was an additional confound that made a true statistical meta-analysis impossible. This limits the generalisability as well as identifying the exact impact of such complex relationship between migraine and anxiety and gender in other settings. The need for a more unified and comprehensive screening tool for anxiety, perhaps a physiological battery of anxiety measures is evident.

## Conclusion

The direction and causes of the migraine comorbidity with anxiety are currently unavailable in the literature. Thus, there is a need for well-designed clinical studies to rigorously identify the clinical characteristics (including the genetic predispositions and neurotransmitter systems) associated with these complex conditions. The outcomes of such studies would lead to a better understanding of the therapeutic strategies for the conditions and design of better management strategies. For example, if anxiety is the trigger for more frequent migraine attacks, then perhaps behavioral treatment strategies targeting anxiety management in males and better hormonal control for females may lead to the desired outcome for migraine management or vice versa.

## Author Contributions

All the authors contributed to the study and development of the paper. All authors have agreed on the final version of the paper and either had: (1) substantial contributions to conception and design (TW, SC, and LK), acquisition of data, or analysis and interpretation of data (LK, LA, and HK) and/or (2) drafting the article or revising it critically for important intellectual content (LK, SC, TW, LA, HK, and AE).

## Conflict of Interest

The authors declare that the research was conducted in the absence of any commercial or financial relationships that could be construed as a potential conflict of interest.
